# Der Einsatz von Servicerobotern bei Epidemien und Pandemien

**DOI:** 10.1365/s40702-020-00669-w

**Published:** 2020-10-14

**Authors:** Oliver Bendel

**Affiliations:** Hochschule für Wirtschaft, Institut für Wirtschaftsinformatik, Fachhochschule Nordwestschweiz, Bahnhofstrasse 6, 5210 Windisch, Schweiz

**Keywords:** Robotik, Serviceroboter, Sicherheitsroboter, Transportroboter, Pflegeroboter, Reinigungsroboter, Wirtschaftsinformatik, Robotics, Service robots, Security robots, Transport robots, Care robots, Cleaning robots, Information management

## Abstract

Seit jeher werden Roboter eingesetzt, um gefährliche oder für uns nicht bewältigbare Aufgaben zu erledigen. Sie entschärfen Bomben, transportieren Gefahrenstoffe und arbeiten sich in für uns nicht erreichbare Gebiete vor. Die COVID-19-Pandemie hat gezeigt, dass auch Serviceroboter, die eigentlich nicht für Sonderfälle vorgesehen sind, hilfreiche Dienste bei der Versorgung von Isolierten und bei der Eindämmung von Krankheiten leisten können. Der vorliegende Beitrag stellt vier Typen von Servicerobotern vor. Dann gibt er Beispiele für die Roboternutzung während der Coronakrise im Jahre 2020. Schließlich wird der Frage nachgegangen, in welchem Umfang und in welcher Weise die Robotertypen zusammenarbeiten können und ob man manche von ihnen zu Generalisten weiterentwickeln kann. Zudem werden Geschäftsmodelle und Betriebsmöglichkeiten thematisiert. Der Beitrag zeigt, dass Kohorten von Robotern in Zukunft lebenswichtig sein könnten.

## Abstract (en)

Robots have always been used to carry out dangerous tasks or tasks that are not manageable for us. They defuse bombs, transport hazardous materials and work their way into areas inaccessible to humans. The COVID-19 pandemic has shown that even service robots, which are not actually intended for special cases, can provide helpful services in the care of isolated persons and in the containment of diseases. This paper presents four types of service robots. Then it gives examples of robot use during the coronavirus crisis in 2020. Finally, the question in which extent and in what way the robot types can cooperate and whether some of them can be developed into generalists is examined. Business models and operating opportunities are also discussed. The paper shows that cohorts of robots could be vital in the future.

## Roboter auf dem Vormarsch

Dass Roboter jenseits der Fabriken – in denen sie vor allem als Industrieroboter auftreten – für außerordentliche Situationen und unter besonderen Umständen eingesetzt werden, ist keine neue Entwicklung. Seit jeher hat man mit ihnen Bomben entschärft, hat sie mit Gefahrenstoffen beladen, ist mit ihnen in Gänge und Tunnel eingedrungen, um Vermisste und Eingesperrte zu finden und zu retten, und hat sie in den Weltraum mitgenommen oder vorausgeschickt. Forschungseinrichtungen arbeiten an autonomen Maschinen für den Kriegseinsatz und für die Vermeidung und Bekämpfung von Waldbränden. Roboter sind, so könnte es ausgedrückt werden, unsere genügsamen, gefühllosen, mit „übermenschlichen“ Fähigkeiten (Tragkraft, Flugfähigkeit, Nachtsicht- und Fernsichtmöglichkeit etc.) ausgestatteten Vertreter.

Mit COVID-19 brach Ende 2019 eine Krankheit aus, die Anfang 2020 zur Pandemie wurde. Wegen der starken Verbreitung und der hohen Ansteckungsgefahr bei SARS-CoV‑2 mussten spezielle Maßnahmen getroffen und auch Neuerungen vorgenommen werden. Die Pandemie hat gezeigt, dass Serviceroboter hilfreiche Dienste bei der Unterstützung von Institutionen, der Versorgung von Isolierten und der Vermeidung von Infektionen leisten können (Zeng et al. 2020; Khan et al. 2020). Zu nennen sind u. a. Sicherheitsroboter, Transportroboter, Pflegeroboter sowie Reinigungs- und Desinfektionsroboter. Vielleicht werden Kohorten von Robotern in Zukunft lebenswichtig sein (Bendel 2020a).

Der vorliegende Beitrag will aufzeigen, welche Serviceroboter in welcher Weise während COVID-19 genutzt wurden, welche Gemeinsamkeiten und Unterschiede und welche Herausforderungen es dabei gab und welche Schlüsse für die Zukunft – insbesondere mit Blick auf ähnliche Krisen und Katastrophen und unter Einbeziehung der Wirtschaftsinformatik – zu ziehen sind. Es soll damit besser verstanden werden, welchen Nutzen Serviceroboter in solchen Situationen stiften und stiften können und wie sich damit der genannten Disziplin neue Betätigungsfelder eröffnen. Im besten Falle können Hochschulen und politische Entscheidungsträger angemessener agieren und reagieren, etwa durch die Erweiterung von Curricula und die Zurverfügungstellung von Ressourcen.

Der Beitrag geht zunächst, nach einer Definition von Servicerobotern, auf die vier genannten Typen ein, die offensichtlich bei Krisen und Katastrophen und speziell Epidemien und Pandemien nützlich sind. Dann werden Beispiele für die Roboternutzung während der Coronakrise gegeben, sodass auch die Frage beantwortet werden kann, ob die genannten Typen tatsächlich Verwendung gefunden haben. Schließlich wird untersucht, wie weit und in welcher Weise die Robotertypen zusammenarbeiten können und ob manche von ihnen Kandidaten für Generalisten sind. Zudem werden Geschäftsmodelle und Betriebsmöglichkeiten thematisiert und damit Perspektiven für die Wirtschaftsinformatik eröffnet. Nicht im Fokus ist die Rolle der Künstlichen Intelligenz (KI) bei der Bekämpfung von SARS-CoV‑2 (Naudé 2020).

## Serviceroboter für Epidemien und Pandemien

Neben Industrie‑, Landwirtschafts‑, Forschungs- und Kampfrobotern sowie Roboterautos sind Serviceroboter eine weitere wichtige Kategorie. Sie sind für Dienstleistungen und Hilfestellungen aller Art zuständig, sie bringen und holen Gegenstände, überwachen die Umgebung ihrer Besitzer oder das Befinden von Patienten und halten ihr Umfeld im gewünschten Zustand (Bendel 2017). Wenn sie mit Sensoren ausgestattet sind, wenn sie über künstliche Intelligenz und Erinnerungsvermögen verfügen, werden sie nach und nach zu scheinbar allwissenden Begleitern, auf jeden Fall zu unangenehmen Auskundschaftern und unbestechlichen Datenträgern. Sie wissen, was ihr Eigentümer oder Gegenüber tut und sagt oder was die Passanten in der Umgebung umtreibt und melden es womöglich an die Hersteller, an die Betreiber oder an Geräte und Computer aller Art.

Offenkundig vollbringen Serviceroboter einen Dienst am Menschen und für Menschen. Sie bieten eben, wie der Name sagt, einen Service, erleichtern das Leben und den Alltag und erweitern, wie viele Roboter, unsere Handlungsfähigkeit (Christaller et al. 2001). Zuweilen werden solche Roboter als Co-Robots oder Cobots bezeichnet, wobei diese Begriffe genauso zunächst für den Kontext der Industrie reserviert werden können. Der klassische Kooperations- und Kollaborationsroboter ist nämlich ein Arm, der eng mit der Arbeitskraft zusammenspannt, sich die Arbeit mit ihr regelrecht aufteilt und sich mit ihr im Arbeitsprozess abwechselt (Buxbaum 2020). Ohne Zweifel können aber Co-Robots in diesem Sinne als Serviceroboter genutzt werden. Ein anderer Begriff in diesem Zusammenhang ist der des sozialen Roboters. Damit werden sensomotorische Maschinen bezeichnet, die für den Umgang mit Menschen oder Tieren geschaffen wurden, wobei sie dafür oft mit animaloiden oder humanoiden Merkmalen sowie mit dem Anschein von Empathie und Emotionen ausgestattet werden (Bendel 2020d). Einige Serviceroboter sind soziale Roboter, andere nicht.

In privaten und (teil-)öffentlichen Bereichen trifft man auf ganz unterschiedliche Typen, auf Sicherheitsroboter, Transportroboter, Informations- und Navigationsroboter, Unterhaltungs- und Spielzeugroboter, Pflege- und Therapieroboter, Reinigungs- und Desinfektionsroboter sowie Gartenroboter. Ob Landwirtschaftsroboter dazuzählen, ist umstritten. Zumindest könnte man manche von ihnen neben die Gartenroboter stellen. Eine andere Systematik begreift Haushalts- und Gartenroboter als eigenen Typ (Bendel 2017) und zählt dazu wiederum bestimmte Reinigungsroboter (vor allem Saugroboter) und Poolroboter. Assistenzroboter oder Companion Robots, die im Haushalt mit Menschen zusammenleben, ihnen ihren Alltag erleichtern und ihnen als Familienmitglied oder Freund dienen, sind eine Vision, die durch Modelle wie Nao und Pepper von SoftBank aus Japan kaum eingelöst wird. Sicherlich sind diese jedoch mehr als Spielzeug- und Unterhaltungsroboter, und sie werden entsprechend auch in anderen Bereichen beschäftigt.

Entscheidend bei Epidemien und Pandemien sind lebensnotwendige Dienste und Aktionen, die für Menschen zu gefährlich oder zu aufwändig wären. Nach Bendel (2020a) bieten sich insbesondere Sicherheits‑, Transport‑, Pflege- und Desinfektionsroboter an – er zieht sowohl bisherige Erfahrungen in der Praxis als auch grundsätzliche Überlegungen heran. Auf diese Typen wird im Folgenden näher eingegangen, wobei Desinfektionsroboter als Verwandte der Reinigungsroboter betrachtet werden. Daneben werden in Zukunft die genannten Assistenzroboter oder Companion Robots von Bedeutung sein, und man könnte Voicebots und Chatbots dazu entwickeln und verwenden, Einsamkeit und Orientierungslosigkeit abzumildern. Khan et al. (2020) führen noch Rezeptions‑, Telemedizin- und Operationsroboter an, wobei letztere vor allem bei Kriegen und Umweltkatastrophen relevant erscheinen, und tatsächlich sind sie im militärischen Kontext entstanden, haben sich dann aber im zivilen Sektor etabliert.^1^

### Sicherheitsroboter

Sicherheitsroboter verbreiten sich auf dem Land wie in der Luft (Bendel 2020c). Sie entlasten oder ersetzen Sicherheitskräfte und sorgen für die Sicherheit der Unternehmen, Besucher und Kunden, etwa indem sie Auffälligkeiten melden oder für Abstand sorgen, wobei sie zugleich das Gefühl von Unsicherheit hervorrufen können. Sie sind autonom beziehungsweise teilautonom oder werden von Menschen oder weiteren Systemen zu Einsatzorten und Problemfällen navigiert und dort für ihre Aufgabe aktiviert. Je nach Zusammenhang werden sie auch als Überwachungsroboter oder Polizeiroboter bezeichnet. Manche von ihnen können mit Hilfe von Kameras (zuweilen Nachtsichtgeräten) und Mikrofonen „sehen“ und „hören“, einige zudem „riechen“, d. h. Gefahrenstoffe und Rauchentwicklung wahrnehmen.

Sicherheitsroboter wie der K3 und der K5 von Knightscope aus Kalifornien sind für den Außen- und den Inneneinsatz geeignet. Der K5 der vierten Generation kann selbst schwieriges Gelände bewältigen – diesbezüglich sind die meisten Sicherheitsroboter defizitär. Genutzt werden sie u. a. im Silicon Valley. Auch in Dubai wird mit ihnen experimentiert, etwa mit einem angepassten REEM von Pal Robotics (Spanien), wie sich überhaupt arabische sowie asiatische Länder interessiert an solchen Technologien zeigen (Bendel 2020c). Diese erweisen sich als heikel in Bezirken, in denen bereits durch Fußgänger und Fahrradfahrer eine hohe Komplexität und eine gewisse Kollisionstendenz vorhanden sind. Die fliegenden Varianten, also Sicherheits- und Überwachungsdrohnen, können den Flugverkehr und Vogelschwärme gefährden, zudem bei einem Absturz Autos treffen und Menschen verletzen oder töten.

Bei Epidemien und Pandemien können Sicherheitsroboter Menschenansammlungen (die ein Sicherheitsrisiko darstellen) erkennen, einzelne Personen auf diese aufmerksam machen oder an eine Maskenpflicht und andere notwendige Verhaltensweisen erinnern. Sie können Gebiete und Räume überwachen und abschirmen, sodass etwa bei einer Quarantäne niemand hinaus und hinein kann. Nicht zuletzt ist es möglich, sie eine Virenkonzentration in Räumen und eine Erkrankung von Personen feststellen und dann Maßnahmen zum Schutz treffen zu lassen.

### Transportroboter

Transportroboter befördern Gegenstände aller Art von einem Beteiligten (etwa dem Anbieter oder Vermittler) zu einem anderen (Kunde, Interessierter, Betroffener) oder begleiten und entlasten Fußgänger und Fahrradfahrer (Bendel 2020b). Sie sind (teil‑)autonom oder werden von Menschen oder weiteren Maschinen von Ort zu Ort gelenkt und dort be- oder entladen. Sie haben mehrheitlich ein Fassungsvermögen von 5 bis 20 Litern, sind also eher auf kleinere Güter ausgerichtet. Je nach Zusammenhang werden sie auch als Liefer- oder Paketroboter bezeichnet. Man kann sie ferner als Industrieroboter sehen, wenn sie in der Fabrik tätig sind, unterwegs mit passenden Komponenten auf vorbestimmten Spuren.

Über Jahre wurden in Europa kleine Transportroboter von Starship Technologies (Kalifornien) erprobt, die für den Außenbereich bestimmt waren, etwa für die Paketzustellung. Sie erwiesen sich als problematisch in Städten, in denen bereits eine hohe Komplexität und eine gewisse Stolper- und Kollisionstendenz vorhanden sind, und mussten streckenweise manuell gesteuert werden (Bendel 2020b).^2^ In Gebäuden sind teils größere Modelle wie Relay von Savioke aus Kalifornien zu finden, bei denen keine Stolper-, allenfalls eine Kollisionsgefahr besteht. Manche (wie wiederum Relay) generieren beim erstmaligen Befahren der Räume und Gänge selbstständig ein 3D-Modell, das von Anwendern einfach modifiziert und konkretisiert werden kann. So ist es möglich, Punkt-zu-Punkt-Verbindungen für übliche Transportwege vorzuschreiben.

Solche Transportroboter eignen sich u. a. für Dienste in Pflegeheimen, Krankenhäusern und Hotels, eingeschränkt auch an Flughäfen. Dort können sie – was bei Epidemien und Pandemien eine wichtige logistische Anforderung ist – Pakete, Nahrungsmittel, Verbandszeug, Laborproben und Medikamente hin und her befördern, ohne dass diese dabei auf ihrem Weg mit Menschen in Berührung kommen. Größere Roboter könnten wiederum Personen befördern, ohne dass dabei Fahrer notwendig wären.

### Pflegeroboter

Pflegeroboter unterstützen menschliche Pflegekräfte und stehen -bedürftigen in unterschiedlicher Hinsicht zur Verfügung (Bendel 2016). Sie bringen und reichen die benötigten Medikamente und Lebensmittel, helfen beim Hinlegen und Aufrichten oder alarmieren den Notdienst (Becker 2018; Bendel 2018). Einige haben natürlichsprachliche Fähigkeiten, sind intelligente und lernende Systeme. Manche Patienten bevorzugen Maschinen gegenüber Menschen bei bestimmten Tätigkeiten, etwa Waschungen im Intimbereich. Andere Tätigkeiten, vor allem sozialer Art, scheinen ungeeignet für Pflegeroboter zu sein (Bendel 2018). Allerdings werden diese mehr und mehr als soziale Roboter konzipiert und können zumindest in Übergangszeiten menschliche Kontakte unterstützen und ersetzen. Der Begriff „Roboter in der Pflege“ zielt nicht nur auf Serviceroboter, die speziell für Pflege und Betreuung entwickelt wurden, eben Pflegeroboter, sondern auch auf Reinigungs- und Transportroboter, die in diesem Bereich genutzt werden können (Bendel 2020d).

Beispiele für Prototypen und Produkte sind JACO, Care-O-bot, Robear, P‑Rob und Lio. JACO, ein Arm samt Hand mit drei Fingern, kann alles in Griffnähe besorgen, Care-O-bot, ein mobiler Assistent des Fraunhofer IPA, sogar alles aus dem Nebenraum (Bendel 2020d). Robear von Riken aus Japan, der an einen Teddy erinnert, hebt Patienten hoch und lagert sie zusammen mit einem Pfleger um – der Protoyp wird im Moment nicht weiterentwickelt. P‑Rob von F&P Robotics aus der Schweiz ähnelt JACO, hat aber lediglich zwei Finger. Er kann sowohl in der Pflege als auch in der Therapie seine Funktion erfüllen. Weitere Modelle des Unternehmens sind Lio, ein mobiler Roboter mit einem Arm, der Flaschen öffnen und Patienten für Termine einsammeln kann (Bendel et al. 2020), und P‑Care, ein mobiler, animaloider Roboter mit zwei Armen, getestet bisher vor allem in China vom dort ansässigen Partner. Sie basieren auf industrieller Cobot-Technologie. Dies scheint vielversprechend für die physischen Funktionen von Pflegerobotern zu sein, während bei anderen Ansätzen nur selten die notwendige Kraft, Geschicklichkeit und Genauigkeit erreicht werden.

Bei Epidemien und Pandemien können Pflegeroboter hochinfektiöse Patienten mit Medikamenten und Lebensmitteln versorgen. Sie können damit auch Pflegekräfte schützen, die mit diesen nicht in direkten Kontakt treten müssen. Sie können zudem Pflegebedürftige versorgen, die zu schwach zum Aufstehen sind. Um Tätigkeiten wie Waschen und An- und Ausziehen erledigen zu können, müssen weitere Fortschritte gemacht werden. Das Spritzen von Substanzen und das Durchführen von Tests wäre ebenfalls eine Option, die z. T. schon realistisch ist.

### Reinigungs- und Desinfektionsroboter

Reinigungsroboter sind (teil-)autonome Roboter. Sie helfen zum einen bei der Nassreinigung von Flächen (Wischroboter) und Fenstern (Fensterputzroboter). Sie setzen dabei Walzen und Bürsten sowie Reinigungsmittel wie Wasser und Chemikalien ein. Bei Poolrobotern ist das Wasser in der Regel im Becken selbst vorhanden. Es wird aber kaum beeinträchtigt. Zum anderen helfen Reinigungsroboter bei der Trockenreinigung von Böden, vor allem als Saugroboter, wie sie im Haushalt vorzufinden sind (deshalb sind diese gängige Haushaltsroboter), oder als Kehrroboter für Straßen und größere Flächen in und vor Gebäuden. Einige Modelle kombinieren Verfahren, können beispielsweise scheuern und saugen. Desinfektionsroboter befreien Flächen von Bakterien und Viren, bestimmte Modelle – unter anderem mit Sprühtechnik und UV-Licht – sogar Möbel, Sanitäranlagen, Türklinken und Knöpfe (Khan et al. 2020).

Mit dem Cleanfix RA660 Navi von Schöler Fördertechnik, dem Adlatus CR 700 von Adlatus Robotics und dem KEMARO-800 von KEMARO sind nur drei Geräte aus dem deutschsprachigen Raum zur Nassreinigung genannt. Manche dieser Geräte sind zusätzlich zur Desinfektion geeignet, allerdings in der Regel nicht von Touchscreens, Türgriffen oder Knöpfen. Beispiele für Poolroboter sind Dolphin E20 von Dolphin und VorteX OV 3505 von Zodiac Poolcare. Auch Saugroboter sind in einer großen Vielfalt vertreten, wenn man an Modelle von iRobot, Roborock, Kärcher, Dyson, SoftBank und vielen anderen Firmen denkt. Der Kehrroboter Blitz One der Berliner Firma Enway hält Gebiete der Hauptstadt sauber (Knoblach 2019), der Schweizer Cleanfix RA660 Navi wurde an Bahnhöfen der Eidgenossenschaft getestet (Schüepp 2017).

Bei Epidemien und Pandemien können Reinigungsroboter etwa Böden von Wohnungen, Bahnhöfen, Flughäfen und Krankenhäusern und Pflegeheimen reinigen, was die Ansteckungsrisiken für das Reinigungspersonal und die Bewohner und Kunden reduziert. Spezielle Modelle sind auch für Türklinken, Fenstergriffe, Möbel etc. tauglich. Mit Desinfektionsrobotern kann man gezielt Bakterien und Viren eliminieren, ob sie sich auf Oberflächen, in der Luft oder auf Lebewesen befinden, und dadurch wiederum Gefahren mindern.

## Serviceroboter während der COVID-19-Pandemie

Die COVID-19-Pandemie hat der Servicerobotik einen Schub verliehen (Yang et al. 2020; Bendel 2020a). Es wurden einige Projekte initiiert, neue Funktionen konzipiert und neue Anwendungsmöglichkeiten ausprobiert. Manche Unternehmen konnten kurzfristig ausgeschriebene Förderungen beanspruchen. Der Einsatz geschah in unterschiedlicher Weise, und er erschien den Betroffenen entweder sachgerecht und zielführend oder aber überzogen (Novak 2020). Die Medien präsentierten die Serviceroboter einerseits als Helfer und Retter, andererseits als Zerstörer von Freiheit und Selbstbestimmung (Rohwedder 2020).

Die Frage ist nun, wie Serviceroboter konkret während der Pandemie eingesetzt wurden. Herrschten die vorgestellten Typen oder andere Arten vor? Wurden andere Arten im üblichen Sinne verwendet oder im Sinne der genannten Typen? Welche Form oder Gestaltung wurde gewählt? All dies soll durch die folgenden Beschreibungen von Fällen aus dem Frühjahr und Frühsommer 2020 deutlicher werden. Gesucht wurde mit Hilfe von Fachdatenbanken und Internetrecherchen, wobei Begriffe wie „COVID-19“, „SARS-CoV-2“, „Corona“, „Service Robot“, „Robot“ und „Drone“ (sowie die deutschen Entsprechungen) kombiniert wurden. Da der Verfasser mehrere Blogs betreibt, war er seit Anfang des Jahres laufend auf Meldungen (etwa in Magazinen wie Gizmodo und Business Insider) gestoßen, die ebenfalls berücksichtigt wurden.

- Ein Roboter von Boston Dynamics erinnerte in einem Park in Singapur die Besucher daran, dass sie die Richtlinien zu Social Distancing befolgen sollten. Spot, so sein Name, ist nicht als Sicherheitsroboter konzipiert, wie der K3 oder der K5. Aber er hat andere Qualitäten: Er kann auf vier Beinen gehen und ist sehr schnell. „Spot was made available for purchase by businesses and governments last year and has specially designed cameras to make sure it doesn’t run into things“ (Novak 2020). Laut einer Pressemitteilung der Singapurer Agentur GovTech waren die Kameras nicht in der Lage, bestimmte Personen zu verfolgen oder zu erkennen, und es sollten keine persönlichen Daten gesammelt werden (Novak 2020). Letztlich wird hier ein Roboter, der eher für militärische Szenarien geeignet erscheint, zu einem zivilen Sicherheitsroboter gemacht. Er dürfte durch seine Gestaltung und die Ruckartigkeit der Bewegungen wie andere Modelle des Unternehmens, das zu SoftBank (Japan) gehört, eher bedrohlich wirken. Als sozialer Roboter, mit anderen Worten, ist er nur bedingt geeignet.
- In China wurden Personen, die ohne Schutzmaske aus dem Haus gingen, über Drohnen aufgespürt und gemaßregelt. Es handelte sich wohl um vereinzelte Aktivitäten, die aber von der Partei durchaus gebilligt wurden (Rohwedder 2020). Die Videos wurden z. T. nachträglich manipuliert, etwa mit Soundeffekten hinterlegt, und bilden nicht unbedingt die Wirklichkeit ab. Bei solchen Einsätzen kommen in der Regel ferngesteuerte Quadrokopter zum Einsatz, wobei festgelegte Strecken automatisch geflogen werden können. Die Stimme ist live, wie von Bahnhöfen der USA bekannt, oder aus der Konserve. Die Reaktionen auf die Videos waren weltweit unterschiedlich. „Während die einen es als weiteres Indiz für den übergriffigen totalen Überwachungsstaat sehen, glauben andere, dass es eine sinnvolle Maßnahme zur Eindämmung der Corona-Epidemie ist“ (Rohwedder 2020). Einige empfanden die Drohnen als direkte Bedrohung, wie man an den Mienen der Ertappten ablesen kann. In diesem Fall wurde eine Fotodrohne mit Lautsprecher als Sicherheitsroboter genutzt.
- Das chinesische Start-up CloudMinds kam Anfang März 2020 in einer kurzfristig aufgebauten Klinik in Wuhan zum Einsatz. „Ein gleichwertiger Ersatz für menschliche Mitarbeiter waren die sechs verschiedenen Robotermodelle zwar nicht. Doch zumindest die Routineaufgaben, die auf dieser Station anfielen, konnten sie dem überarbeiteten Krankenhauspersonal abnehmen: das Verteilen von Medikamenten oder Essen, das Einsammeln von Abfällen und benutzter Bettwäsche, die Reinigung und Desinfektion einzelner Bereiche oder die Identifikation von Patienten mit erhöhter Temperatur“ (Kerler 2020). Bill Huang, der CEO, wurde von BBC mit den Worten zitiert: „This is China’s first-ever entirely robot-led ward and an opportunity to test the capability of the technology and how we work together“ (BBC 2020). Die Gestaltung der Maschinen war ganz unterschiedlich: „Manche der eingesetzten Roboter, der CloudPepper etwa, sehen eher menschlich aus und sollen mit kleinen Tanzeinlagen zwischendurch für ein bisschen Unterhaltung in der Quarantäne-Station gesorgt haben. Andere ähneln eher rollenden Boxen“ (Kerler 2020). Das Unternehmen vertraut auf cloudbasierte, lernfähige Systeme, die in ganz unterschiedlichen Kontexten reüssieren können. Dabei werden eben handelsübliche Roboter wie Pepper eingebunden. So war nebenbei die soziale Robotik mit im Spiel.
- In Shenyang im Nordosten Chinas überprüfte ein Roboter die Temperaturen der Besucher, um dem Krankenhauspersonal zu helfen und weitere Infektionen zu verhindern (BBC 2020). Solche Fähigkeiten rückten während der Coronakrise einige Projekte in den Vordergrund, wobei ganz unterschiedliche Verfahren verwendet wurden (die nicht allesamt auf Roboter angewiesen sind). Ein Krankenhaus in Indien stellte den humanoiden, sozialen Roboter Mitra an, um mittels einer Wärmebildkamera mögliche Corona-Patienten zu erkennen. Erst nach der Untersuchung wurden diese an das Pflegepersonal weitergeleitet (Meisenzahl 2020a). Nach einem Bericht derselben Autorin verwendete eine Apotheke in Turin Roboter, um bei Kunden das Fieber zu messen und das Tragen von Masken zu überprüfen (Meisenzahl 2020b). Auf den Fotos ist allerdings ein großes Handy oder kleines Tablet mit einem Aufsatz zu sehen, wohl der Wärmebildkamera. An der University of Southern Denmark wurde ein Roboterarm für weitergehende, sozusagen echte COVID-19-Tests ausprobiert (Dalgaard 2020). Die Patienten mussten für den Rachenabstrich ihren Kopf in ein Gestell legen, das man von Arztpraxen her kennt.
- Auch Desinfektionsroboter waren weltweit im Einsatz. „Mehrere chinesische Hospitäler wollen auf die selbstfahrenden Desinfektionsroboter des dänischen Start-ups UVD Robots setzen. Diese zerstören mithilfe von ultraviolettem Licht die DNA oder RNA von Mikroorganismen wie Viren, die sich auf Oberflächen eingenistet haben. Seit Jahren wird UV-Licht zu diesem Zweck eingesetzt – zuvor nur nicht in Verbindung mit Robotern“ (Kerler 2020). In Hongkong finden sich laut der Quelle kleinere, kastenförmige Roboter, um die U‑Bahn zu entkeimen. Auch der Technologiekonzern Siemens entwickelte nach Angaben des Magazins in einer chinesischen Dependance einen ferngesteuerten Desinfektionsroboter, der „klassisches“ Desinfektionsmittel verteilt. Der Flughafen Hongkong präferierte gleichfalls automatisierte Konzepte und testete Ganzkörperdesinfektionskabinen. Daneben kamen die eher dinghaft gestalteten Whiz von SoftBank und ISR von TMiRob zum Einsatz, die Bakterien und Viren aus der Luft filtern und mit UV-Licht von Flächen entfernen können (Asaf 2020).
- Im deutschsprachigen Raum wurden mehrere Projekte lanciert und Roboter aufgeboten, vor allem wiederum im Bereich der Reinigung und Desinfektion. Der Tages-Anzeiger titelte am 28. Mai 2020: „Flugpassagiere bewegen sich zwischen Putzrobotern und Maskenautomaten“. Erwähnt wurden Maßnahmen am Flughafen Zürich. „Zwei Roboter stehen im Einsatz, die den Boden putzen. Das schützt zwar nicht vor Covid, gibt den menschlichen Reinigungskräften allerdings mehr Zeit zur manuellen Reinigung von Kontaktflächen“ (Staehelin 2020). Damit werden die Geräte nicht direkt zur Bekämpfung der Pandemie eingesetzt, aber indirekt. Ab Sommer 2019 wurde bereits der Bodenreinigungsroboter cleanfix RA 660 Navi getestet. Bei den neuen Robotern handelt es sich um andere Modelle, in der Art eines Taski Intellibot 2000 von Diversey, die mit aufgemalten Augen sowie einem aufgepinselten Blaumann ausgestattet und somit anthropomorphisiert wurden. Ob man mit den sozialen Robotern am Flughafen um Sympathien werben will, ist nicht bekannt.
- DIH-HERO ist ein Projekt im Gesundheitssektor, das seit Januar 2019 von der Europäischen Union unterstützt wird. Laut Website besteht die Aufgabe darin, ein tragfähiges Netzwerk zu schaffen, das die Akteure im Gesundheitssektor miteinander verbindet, und kleine und mittlere Unternehmen zu unterstützen. „Currently, Europe and countries all over the world are facing a global pandemic. Together with its extensive Robotics in Healthcare European network DIH-HERO decided to support the fight against COVID-19 by providing € 1,000,000 for robotic technologies that can be deployed timely, in order to support healthcare professionals and save lives by satisfying a current clinical demand or need“ (DIH-HERO o. J.). F&P Robotics war einer der Gewinner der Ausschreibung. Lio durfte neue Aufgaben im Bereich der Desinfektion (Türfallen, Liftknöpfe) erlernen. Mit ihm wurde ein Industrieroboter (ein Cobot) zu einem Serviceroboter umfunktioniert und nun zu neuen Aufgaben befähigt. In Bezug auf seine Akzeptanz liegen schon mehrere Studien vor (Wirth et al. 2020; Früh und Gasser 2018; Bendel et al. 2020) – im Allgemeinen wird er von den Pflegebedürftigen gut aufgenommen, umso besser, je höher der Nutzen für sie ist, etwa wenn er etwas aufhebt, reicht oder öffnet. Wie die neuen Aufgaben akzeptiert werden, wird zu untersuchen sein.

Wie deutlich wurde, tauchen die genannten Typen in der Praxis durchweg auf. Allerdings fällt auf, dass einige von ihnen zweckentfremdet oder eher generalistische Roboter zu spezifischen Aufgaben herangezogen werden. Dies mag unterschiedliche Gründe haben. Sicherlich ist vor Ort nicht stets der richtige Roboter verfügbar. Zudem will man – dies wurde bei China augenfällig – die eigene Schlagkraft und Innovationsfreudigkeit beweisen und Produkte promoten, die im eigenen Land hergestellt wurden, selbst wenn es wie bei Siemens um „fremde“ Firmen geht. Zu bemerken ist, dass es sich weltweit um überschaubar viele Anwendungen mit nur wenigen Robotern gehandelt hat und bei der Recherche vor allem Europa und Asien aufgetaucht sind, weniger die USA, Kanada, Australien und Afrika.^3^ Die Liste ist dennoch keineswegs abschließend, und es ist nochmals zu betonen, dass die Coronakrise der Servicerobotik einen Schub verliehen hat.

## Der Einsatz von Servicerobotern in der Zukunft

Der Einsatz von Servicerobotern steckt in den Kinderschuhen, hinsichtlich ihrer Leistungsfähigkeit, ihrer Verbreitung und Verfügbarkeit und mit Blick auf spezielle Situationen wie Epidemien und Pandemien. Es müssen u. a. Überlegungen angestellt werden zu ihren Zusammenarbeits- und Entwicklungsmöglichkeiten. Zudem ist nach Geschäftsmodellen und Betriebsmöglichkeiten zu fragen, ein Thema nicht zuletzt für die Wirtschaftsinformatik.

### Kooperation, Kollaboration oder Generalismus

Die behandelten Roboter waren mehrheitlich als singuläre Systeme unterwegs, mit vorab definierten Aufgaben. Einzig CloudMinds hat die Roboter in eine Infrastruktur eingebettet und eine Vernetzung der Roboter im Sinn gehabt. Dieser Ansatz scheint interessant zu sein, um sie effizienter und effektiver zu machen. Es könnte eine Kooperation und Kollaboration nicht bloß zwischen Robotern und Menschen entstehen, sondern auch zwischen Robotern. Diese würden sich gegenseitig helfen, sich gegenseitig behandeln und instand setzen und nach Möglichkeit voneinander lernen. Dabei spielt auch die kollektive Intelligenz (engl. „collective intelligence“) eine Rolle (Mavridis et al. 2012; Gavriushenko et al. 2020), unter Beteiligung natürlicher wie künstlicher Akteure und Aktanten (Latour 2007). Im Folgenden werden Beispiele für eine solche Kooperation und Kollaboration genannt, auf der Grundlage von eigenen Überlegungen, die wiederum auf den genannten Darstellungen und eigenen Beobachtungen beruhen.

- Transportroboter befördern Medikamente, Verbandsmaterial und Nahrungsmittel zu Verletzten und Kranken. Nachdem diese die Güter entnommen haben, rollen die Roboter zurück und werden von Reinigungs- und Desinfektionsrobotern empfangen, die sie für den nächsten Einsatz vorbereiten. Pflegeroboter sind nach getaner Arbeit entsprechend zu präparieren.
- Mehrere Transportroboter sind in der Lage, schwere Güter zu transportieren, etwa kleinere Gebäudeteile zur Errichtung von Pop-up-Kliniken oder Container mit Fäkalien. Sie nehmen diese gemeinsam auf, verteilen die Last und bringen sie zu einem bestimmten Ort, wo die Entladung stattfindet. Montage- oder Entsorgungsroboter mögen die weitere Arbeit übernehmen.
- Mehrere Pflegeroboter sind beim Umbetten und Aufrichten hilfreich – wenn dies von einem Modell geleistet werden muss, muss dieses sehr schwer und stabil sein, wie Robear, wobei dann Gefahren durch Umkippen und Herunterfallen (bei Treppen) entstehen. Sie wechseln sich gegenseitig ab, wenn sie Strom benötigen und dringende Dienste erledigt werden müssen.
- Sicherheitsroboter kooperieren als Unmanned Aerial Vehicles (UAV), also Drohnen, und Unmanned Ground Vehicles (UGV), also Bodenfahrzeuge und Landroboter. Beispielsweise fliegen die einen den anderen voraus, um die Situation zu erkunden, und dann wird – von Maschinen oder Menschen – entschieden, ob die Fahrzeuge folgen oder halten sollen.
- Reinigungsroboter bringen Pflegerobotern die Reinigung von Flächen, Griffen etc. bei, indem sie Reinigungsmittel vorschlagen, Bewegungen vorführen und Unterschiede von Materialien erklären. Gerade Bewegungen stellen eine Herausforderung dar, weil Reinigungsroboter recht kompakte, geschlossene Systeme sind, Pflegeroboter dagegen oft Arme und Greifer haben.

Vorbilder für solche Konstellationen sind durchaus vorhanden. So ist die UAV-UGV-Kombination eine Methode, um Rehkitze im Feld rechtzeitig zu erkennen und zu retten (Israel et al. 2010), wobei das Fahrzeug genauso ein konventioneller Mähdrescher sein kann und in diesem Fall das Bordsystem (oder ein Smartphone) und am Ende der Fahrer die Information erhält. Die Gestaltung als sozialer Roboter hat nicht nur Konsequenzen für die Zusammenarbeit mit Menschen, sondern auch mit Maschinen, insofern häufig Arme, Hände, Augen und andere animaloide oder humanoide Elemente vorhanden sind.

Roboter können demnach, wie nicht nur dieser Fall zeigt, sowohl mit anderen Robotern als auch mit anderen Geräten und Infrastrukturen kommunizieren und interagieren. Das erinnert an den Automobilbereich. So sind Module für die Car2Car oder Car2X Communication bereits enthalten, die dann beim hoch- und vollautomatisierten beziehungsweise autonomen Fahren ihr Potenzial entfalten. Letztlich sind stark automatisierte und autonome Fahrzeuge nichts anderes als Roboter. Das X kann zum Beispiel für ein Verkehrsleitsystem oder eine verchippte Person stehen. Bei Servicerobotern werden solche Verfahren vereinzelt angewendet. So sind einige, wie deutlich wurde, an Cloud-Anwendungen angebunden, durch die sie spezifische Informationen erhalten oder in größerem Umfang lernfähig sind. Auch kommunizieren manche Roboter – wie Produkte von Jinn-Bot Robotics & Design – mit Kameras und anderen Sensoren, um bessere Einblicke in ein Gebäude respektive eine Situation und eine Außenansicht von sich selbst zu erhalten. Dies erinnert wiederum an die Roboselfies von Raum- und Bodenfahrzeugen, die den Ingenieuren nützlich beim Feststellen von Schäden sind (Bendel 2014).

Eine gewisse Alternative zu Kooperation und Kollaboration wäre, dass einzelne Roboter zu Generalisten werden. In dieser Richtung wurden Anfänge sichtbar. So ist Lio eigentlich ein Roboter, der in Pflege und Betreuung seine Stärken hat. Dennoch konnte das EU-Projekt davon überzeugt werden, dass er für die Desinfizierung genutzt werden kann. Wie bereits erwähnt, handelt es sich im Grunde um einen mobilen Co-Robot aus der Industrie, dem alles beigebracht werden kann, was mit Halten, Greifen, Öffnen etc. zu tun hat. Man kann einen solchen auch neben einem Bett postieren und von Gedanken steuern lassen – Querschnittsgelähmte können davon ebenso profitieren wie Menschen, die vorübergehend ihren Körper nicht unter Kontrolle haben (Podbregar 2018). Dennoch ist eine Fremddesinfizierung unter Umständen zielführender, und auch Schwärme und Herden können sinnvoller sein, wie bei den beschriebenen Schwertransporten, bei denen – etwa mit Hilfe der kollektiven Intelligenz – eine Kooperation, Kollaboration und Koordination der Roboter sowie eine Verteilung der auf ihnen ruhenden oder von ihnen angehobenen Last stattfindet.

### Geschäftsmodelle und Betriebsmöglichkeiten

Sicher sollten Unternehmen nicht eine größere Anzahl von Servicerobotern auf Halde produzieren, zumal sich die Anforderungen je nach Krise und Katastrophe ändern können. Sinnvoll wären garantierte Abnahmen durch staatliche und private Einrichtungen mit bestimmten Serviceverträgen. Um hier das Risiko nicht zu sehr auf die Anwender zu übertragen, sind flexible Konzepte notwendig. Vorbild könnte wiederum das teil- und hochautomatisierte Fahren sein. Tesla-Autos haben eine komplexe IT-Infrastruktur und Hardware, die im Prinzip viele Anforderungen abdecken kann. Sie werden sozusagen über Nacht über ein Softwareupdate modifiziert (Ingle und Phute 2016). So könnten sie sich in Zukunft innerhalb von Minuten oder Stunden in ein autonomes Auto verwandeln. Ähnlich könnte bei Servicerobotern verfahren werden.

Flexibilität in Bezug auf das Gehäuse des Roboters, die Arme, Beine oder Rollen etc. gewinnt man durch 3D-Druck. Schon heute setzen verschiedene Firmen darauf, etwa Jinn-Bot und Savioke. Nicht nur kann man so schnell sich ändernde Bedürfnisse von Abnehmern befriedigen – diese können womöglich selbst benötigte Teile vor Ort nachdrucken und sich so von Lieferanten unabhängig machen. Sie benötigen lediglich den Bauplan eines Teils, der über E‑Mail oder über B2B-Plattformen zu übermitteln ist. Modulare Konzepte helfen dabei, dass verschiedenartige Roboter auf derselben Basis gebaut werden können, ein Prinzip, das die Automobilindustrie seit langem praktiziert und perfektioniert hat.

Es ist nicht immer voraussehbar, wo sich Krisen und Katastrophen ereignen oder wie sich Epidemien ausbreiten (und ob oder wie schnell sie zu Pandemien werden). Um in einem Gebiet möglichst viele passende Roboter bereitzustellen, die zudem kooperieren und kollaborieren können, braucht es geeignete Betriebskonzepte. Denkbar ist, dass jede Stadt oder jeder Bezirk eine Mindestversorgung mit Robotern hat, ähnlich wie eine Feuerwehr oder eine Stromversorgung existieren muss. Der 3D-Druck kommt hier erneut ins Spiel. Wenn die Baupläne und die technischen Möglichkeiten vorhanden sind, können Roboter recht schnell am Brennpunkt produziert und repariert werden. Dabei mögen Kooperationspartner der Hersteller helfen.

Die Energieversorgung selbst ist eine Herausforderung, denn nicht allein Roboter benötigen Strom, sondern auch die damit verbundenen Cloud-Dienste und KI-Anwendungen. Freilich findet deren Betrieb in der Regel woanders statt und ist beliebig skalierbar. Zudem benötigen Serviceroboter Raum, bei der Lagerung wie im Betrieb. Dies geht zuweilen vergessen: Sie sind unsere Assistenten, aber auch unsere Konkurrenten, und gerade Städte bieten nicht beliebigen Raum. Es muss entsprechenden Vorbehalten der Bevölkerung Rechnung getragen werden. Wenn Serviceroboter überall unter uns sind, könnte sich der Widerstand gegen sie erhöhen, zumal sie als mobile Roboter häufig Kameras und Mikrofone haben und potenzielle Spione sind, sodass Privat- und Intimsphäre gefährdet werden. Es ist sinnvoll, die Gesellschaft in die Entwicklung von Servicerobotern einzubeziehen, über Umfragen und Akzeptanzforschung, sie über aktuelle Entwicklungen aufzuklären – und Technologien, die aus guten Gründen vehement abgelehnt werden, wie Gesichtserkennung, erst gar nicht zu verwenden.

## Sich vermehrende Maschinen

Der vorliegende Artikel eruierte den möglichen Mehrwert von unterschiedlichen Typen von Servicerobotern für Krisen und Katastrophen. Dabei wurde der Frage nachgespürt, wie weit und in welcher Weise die Robotertypen zusammenarbeiten können und ob manche von ihnen Kandidaten für Generalisten sind. Andere Typen sollten in weiteren Beiträgen einbezogen werden, etwa Telemedizinroboter, über die Ärzte Diagnosen erstellen können, oder Rezeptionsroboter, die evtl. bei der Triage helfen. Es zeigt sich, dass Kohorten solcher Maschinen in Zukunft lebenswichtig sein könnten. Sie könnten Teil der Grundversorgung sein und im Bedarfsfall herangezogen werden. Insbesondere Epidemien und Pandemien sind ein realistischer Rahmen, und es steht zu befürchten, dass noch weitere auftreten werden, da kaum Maßnahmen mit Blick auf die Ursachen getroffen wurden.

Man steht sicherlich noch ganz am Anfang, und wenn man die Szenarien zu Kooperation und Kollaboration kritisch betrachtet, wird man diagnostizieren, dass im Moment weder Software noch Hardware der Geräte den Anforderungen gewachsen sind und deren Zusammenarbeit an Grenzen stößt. Genau hier braucht es weitere Forschung – eine erfolgreiche Kooperation und Kollaboration zwischen Robotern und zwischen Robotern und Menschen gestaltet sich nicht einfach. Die Künstliche Intelligenz versetzt die Maschinen in die Lage, sich gegenseitig bestimmte Fähigkeiten beizubringen. Die Wirtschaftsinformatik vermag in Bezug auf Geschäftsmodelle und Betriebsmöglichkeiten ihren Beitrag zu leisten.

Technik‑, Roboter- und Wirtschaftsethik können ebenfalls zur Diskussion beitragen, etwa zur Frage, ob der Einsatz von Servicerobotern im Einzelfall gegen die Menschenwürde verstoßen und ob die menschliche Arbeitskraft im Extremfall dauerhaft verdrängt werden und so die Existenzsicherung gefährdet sein kann. Ein Aspekt, für den die Informationsethik die zuständige Bereichsdisziplin ist, wurde bereits angesprochen, nämlich der der Privat- und Intimsphäre und mithin der informationellen Autonomie – zudem kann sie der Frage nachgehen, ob sich neue digitale Gräben auftun, verursacht durch den möglichen oder nicht möglichen Einsatz digitaler Technologien.

Wenn man weiter in die Zukunft schaut, tauchen weitere Szenarien auf. Die Roboter könnten sich gegenseitig bauen, auch dann, wenn viele Informationen verloren gegangen und stabile Strukturen rar geworden sind. Helfen mag dabei die DNA of Things, die in die Maschinen eingepflanzt werden und den Bauplan enthalten könnte (Koch et al. 2020). Das klingt sehr nach einer Dystopie: Maschinen irren umher, treffen aufeinander und vermehren sich. Aber wenn sie dann in der Lage sind, Menschen zu finden und zu retten, scheint sich das zur Eutopie zu wandeln. Man würde sich freilich wünschen, dass man manche Technologien, so faszinierend sie sind, erst gar nicht bräuchte.

## Footnotes

1 Interessant ist, dass der Markt für Serviceroboter nicht von drei, vier Firmen dominiert wird, sondern sich eine große Vielfalt findet. Auch der deutschsprachige Raum ist gut vertreten, insbesondere bei Reinigungsrobotern.

2 Alternativ oder zusätzlich werden Transportdrohnen (meist Quadrokopter) verwendet.

3 Serviceroboter sind an keinem Ort der Welt massenhaft vorhanden. Genau dies sollte sich unter Umständen ändern, wobei es entsprechende Betriebskonzepte braucht.
